# Is Transcriptomic Regulation of Berry Development More Important at Night than During the Day?

**DOI:** 10.1371/journal.pone.0088844

**Published:** 2014-02-13

**Authors:** Markus Rienth, Laurent Torregrosa, Mary T. Kelly, Nathalie Luchaire, Anne Pellegrino, Jérôme Grimplet, Charles Romieu

**Affiliations:** 1 Fondation Jean Poupelain, Javrezac, France; 2 INRA-SupAgro, UMR AGAP, Montpellier, France; 3 Laboratoire d’Oenologie, UMR1083, Faculté de Pharmacie, Montpellier, France; 4 INRA, UMR LEPSE, Montpellier, France; 5 ICVV (CSIC, Universidad de La Rioja, Gobierno de La Rioja), Logroño, Spain; 6 INRA, UMR AGAP, Montpellier, France; Karlsruhe Institute of Technology, Germany

## Abstract

Diurnal changes in gene expression occur in all living organisms and have been studied on model plants such as *Arabidopsis thaliana*. To our knowledge the impact of the nycthemeral cycle on the genetic program of fleshly fruit development has been hitherto overlooked. In order to circumvent environmental changes throughout fruit development, young and ripening berries were sampled simultaneously on continuously flowering microvines acclimated to controlled circadian light and temperature changes. Gene expression profiles along fruit development were monitored during both day and night with whole genome microarrays (Nimblegen® vitis 12x), yielding a total number of 9273 developmentally modulated probesets. All day-detected transcripts were modulated at night, whereas 1843 genes were night-specific. Very similar developmental patterns of gene expression were observed using independent hierarchical clustering of day and night data, whereas functional categories of allocated transcripts varied according to time of day. Many transcripts within pathways, known to be up-regulated during ripening, in particular those linked to secondary metabolism exhibited a clearer developmental regulation at night than during the day. Functional enrichment analysis also indicated that diurnally modulated genes considerably varied during fruit development, with a shift from cellular organization and photosynthesis in green berries to secondary metabolism and stress-related genes in ripening berries. These results reveal critical changes in gene expression during night development that differ from daytime development, which have not been observed in other transcriptomic studies on fruit development thus far.

## Introduction

The grapevine is one of the most abundant perennial crops in the world with a total surface of approximately 7.6 million hectares planted under vines [1]. Complex, poorly understood processes, occurring at different stages throughout berry development determine the final quality of the fruit. The development of the grapevine berry follows a double sigmoid growth pattern consisting of two distinct growth phases separated by a lag phase [2]. Cell division triggered at anthesis occurs only during the first phase of berry development, which lasts approximately 50 to 60 days after flowering, depending on cultivar and environmental conditions [3], [4]. This stage is marked by a first period of vacuolar expansion that relies on the synthesis and storage of tartaric and malic acid [5] as the major osmoticums at a vacuolar pH of approximately 2.6 [6]. Several other compounds, with an important effect on ultimate wine quality are also accumulated during the first growth period of the berry. Amongst these are hydrocinnamic acids, tannins, amino acids [7], [8], [9] and some aroma compounds such as methoxypyrazines in varietals such as Cabernet Sauvignon, Cabernet Frank and Sauvignon blanc [10], [11]. The first growth phase is followed by a lag phase where berry growth and organic acid accumulation cease. The most significant changes in gene expression are triggered during the 24 h transition phase between the lag phase and ripening, where berries suddenly soften individually [12], [13]. During the subsequent ripening phase, the volume of the berry roughly doubles, with the accumulation of approximately 1 M hexoses as osmoticums, and the respiration of malic acid is induced simultaneously with sugar loading. During ripening, amino acids and anthocyanins accumulate [3] and major aromatic compounds including terpenes, norisoprenoids, esters and thiols are synthesized [10]. The control of these physiological processes is not well understood in the grapevine – which is a non-climacteric fruit exhibiting completely different developmental characteristics from climacteric fruit such as tomato or banana which have been more extensively studied [14].

Since the publication of the grapevine Genome in 2007 [15] several high-throughput technologies have been developed in order to gain a greater understanding of the regulation of physiological changes occurring during berry development. Studies using microarrays or RNA sequencing technology have been carried out on economically important *Vitis Vinifera* L. cultivars, for example Chardonnay, Muscat de Hamburg, Trincadeira, Cabernet Sauvignon, Shiraz, Corvina and Pinot Noir. [13], [16], [17], [18], [19], [20], [21], [22]. These studies led to a greater understanding of some traits of berry ripening including the regulation of tannin and anthocyanin biosynthesis pathways [23]. However, major physiological events such as the onset of malic acid respiration are not fully understood at this time [24], [25]. Presumably the lack of significant transcriptional changes in such studies is due to sampling protocols that did not pay sufficient attention to specific time points during berry development. Other possible reasons are uncontrolled environmental conditions leading to the introduction of significant, unquantifiable biases in gene expression, covering developmentally regulated changes.

All studies on grapevine berry development have been conducted on field grown grapevines where impacts on gene expression arising from environmental conditions cannot be avoided. Furthermore, all studies on berries and other fleshy fruits were carried out during the day. For this reason changes occurring throughout berry development during the night were neglected, despite the knowledge of significant diurnal changes, such as fruit swelling during the nighttime [26], [27], daytime-dependent regulation of photosynthesis [28] and changes in gene expression related to the circadian clocks. The latter, whose central function is to sustain robust cycling across a wide range of light and temperature conditions are known to regulate physiology in order to respond to the day/night cycle [29]. Circadian timing involves the rhythmic expression of genes that were identified in many organisms and tissues from cyanobacteria to mammals [30], [31]. Studies of gene expression by transcriptomics were the first global experiments to provide information on the molecular rhythms at the whole plant level [32]. Early time–course studies estimated that 2–16% of the steady state transcriptome is regulated by the circadian clock with peak phases occurring throughout the day [33], [34]. The circadian effect is well buffered across a range of temperatures and conditions by a compensatory mechanism [35]. This is the first study where gene expression during berry/fleshy fruit development was characterized simultaneously during the day and at night.

The studied microvine is a *GAI1* (GA insensitive) mutant regenerated from the L1 cell layer of Pinot Meunier L., exhibiting a dwarf stature and an early and continuous fructification along the main vegetative axis [36], [37]. It was previously proposed as a new model for grapevine research in genetics and physiology [38], [39], [40] and was shown to be adapted for small scale experiments in climatic chambers [41]. The dwarf stature of the microvine made it possible to grow plants under strictly controlled conditions during the whole period of reproductive development, and to obtain simultaneously, on the same plant, fruits at different developmental stages, thus minimizing the introduction of environmental biases linked to field conditions or noticeable changes in photoperiod during the reproductive cycle. A whole genome approach with Vitis 12X Nimblegen® 30 K microarrays was used on four different developmental stages sampled during the day and night. Results show that developmental regulation of gene expression at night is very critical for grapevine fruit development with many genes responding in a different manner between developmental stages. The number and categories of modulated genes between day and night differ tremendously depending on the different stages of berry development especially between the green and the ripening berry.

## Results and Discussion

### Stage Selection and Validation of Experimental Design

**Figure 1:**
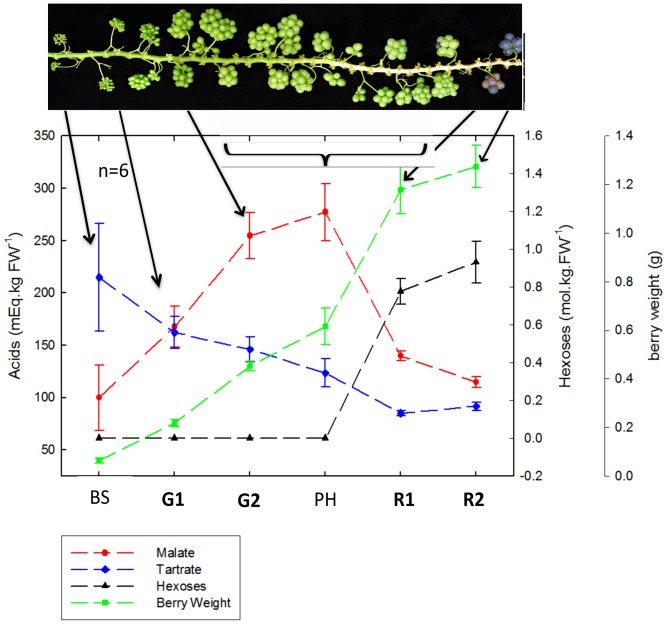
Main biochemical characteristics of sampled berries. BS: Berry Set, G1: Green stage 1, G2: Green stage 2, PH: Plateau Herbacé/lag phase, R1: Ripening stage 1, R2: Ripening stage 2.

Berries at six developmental stages were sampled simultaneously during the day or night: berry set (BS), two stages during green growth (G1, G2), lag phase or “plateau herbacé” (PH) and two ripening phases (R1 and R2; Figure 1). Berries from microvines displayed the same three typical phases of development as field vines in relation to the evolution of fresh weight and major solutes (Figure 1). The first or green growth period where malic acid concentration increases up to 280 mEq is followed by the lag phase with berry growth and acid accumulation leveling off at around 0.6 g berry weight. Thereafter growth is resumed; hexose accumulation starts simultaneously with the breakdown of malic acid, until berry weight reaches 1.4 g and hexoses reach 1 M at maturity. Tartaric acid accumulation ceases at 120 mEq during the first growth period, yielding a malate to tartrate ratio of 2.3, before reducing in concentration due to dilution, while remaining constant on a per berry basis (data not shown).

The amino acid profile of berries is presented in Table S1. The most abundant amino acids of the microvine berry were proline (pro), arginine (arg) followed by alanine (ala), glutamic acid (glu), aspargine (asp), threonine (thr), glutamine (gln) and lysine. Free amino acid concentrations vary depending on cultivar, rootstock/scion combinations, vine nutrient management, vineyard site, and growing season [43]. However, the microvine presents an amino acid profile comparable to field grapevine cultivars [42], [43]. From these observations, it can be concluded that the *gai1* mutation in the dwarf phenotype of the microvine does not impact major fruit developmental features. This can be explained by the tissue specificity of *GAI1* that is expressed in several grapevine organs but not fruits, conversely to other GAIs genes (data not shown).

Four stages were selected for transcriptomic analysis, including two stages in each successive growth period. Berry growth and acid accumulation occurred at maximal rate in G1 and more slowly in G2, just before the lag phase. In the same manner, two stages were selected during ripening, which share quite close physiological characteristics, but with slower growth and sugar import rates in R2 as compared to R1.

Of the 9273 transcripts detected as modulated between at least two stages, (fold change (fc) >2; pval adj <0.05), 7430 of these were simultaneously detected in both day and night samples; 1843 appeared in the night only, whereas none were restricted to day samples (Table S3). This repartition *a posteriori* validates robust changes in gene expression hitherto obtained through day-screenings as reported in the literature [13], [16], [17], [18], [19], [20], [21], [22]. However, a substantial part of developmentally regulated changes in gene expression occurring specifically at night was totally overlooked so far. Transcripts modulated in microvine berries between green and ripening stages were compared with data extracted from Fasoli *et al*., 2012 [44] conducted on *Vitis vinifera* cv Corvina berries, available in the Gene Expression Omnibus under the series entry GSE36128 (http://www.ncbi.nlm.nih.gov/geo/query/acc.cgi?token=lfcrxesyciqgs joandacc = GSE36128).

1970 transcripts were detected in Corvina berries between the stages called “post-fruit set” (green berry) and “ripening”. Of these, 1550 (79%) were also modulated in microvine between green and ripening berries (Table S6) and showed good linear correlation in their expression (R^2^ = 0.72; Figure S5). The large number of commonly modulated genes despite different genotypes, environmental conditions and sampling stages, validates the microvine as a model for the study of berry physiology and transcriptomics. In contrast, it must be emphasized that 94% of the 1843 genes detected here that were specifically modulated during nighttime development have not been observed in daytime experiments on Corvina berries.

**Figure 2:**
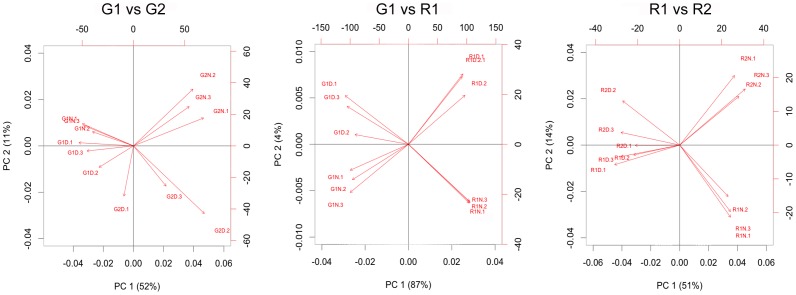
Principal component analysis separately on green stages (left), ripe stages (right) and between green and ripe (middle) during the day and night.

Analysis of the data at each of the four stages revealed that 2684 transcripts changed expression during the day/night transition at one developmental stage at least. Amongst them 1849 (70%) also showed developmental changes between individual growth stages. An overview of down- and up-regulated transcripts between day and night is presented in Table S2. Principal component analysis (PCA; Figure 2) was applied separately on the two green stages, the two ripening stages and between G1 and R1. The two green stages are separated by the first PC explaining half of the variation in gene expression with greater differences for the night samples compared to day samples. The second PC, accounting for 11% of the variation in gene expression represents the day/night axis and shows a clearer separation for G2. The PCA on ripening stages yields an inversion of these axes, with PC1 explaining once again half of the variation but separating day and night, while developmental stages can be distinguished by PC2 (14% variance) for the night samples only. In the plot between G1 and R1 90% variance can be attributed to development (PC1) and only 4% account for day and night differences (PC2). This large variation between green and ripe berries concurs with the fact that most important changes in gene expression occur at the onset of ripening in developing berries [12], [13]. The day/night discrimination explained by PC2 is more pronounced for the later rather than for the earlier developmental stages. These results highlight the importance of considering the berry transcriptome at night where close stages seem to show more significant differences than during the day.

### Developmentally Regulated Gene Expression

**Figure 3:**
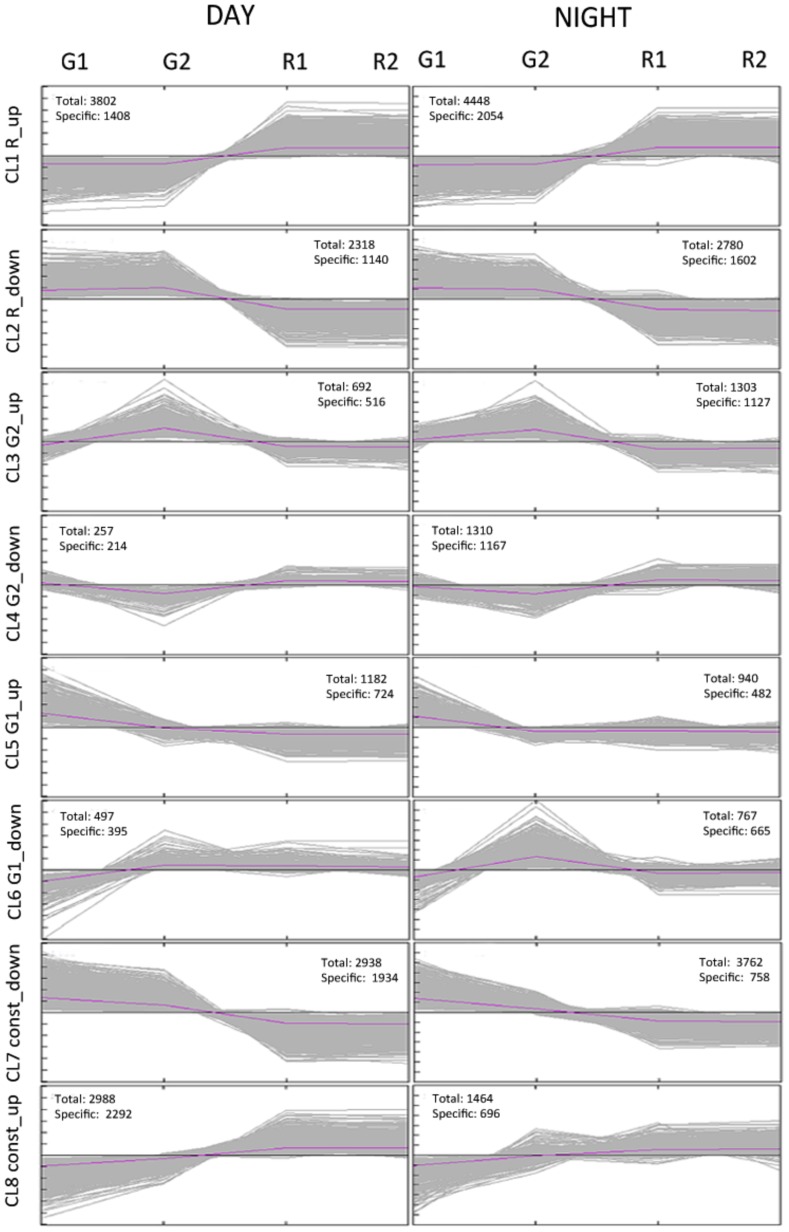
Expression profiles of developmentally regulated genes during the day and night. Clustering was performed using k-means statistics on mean centered RMA normalized expression log_2_ values. Numbers of all day respectively night specific transcripts in each cluster are displayed.

**Figure 4:**
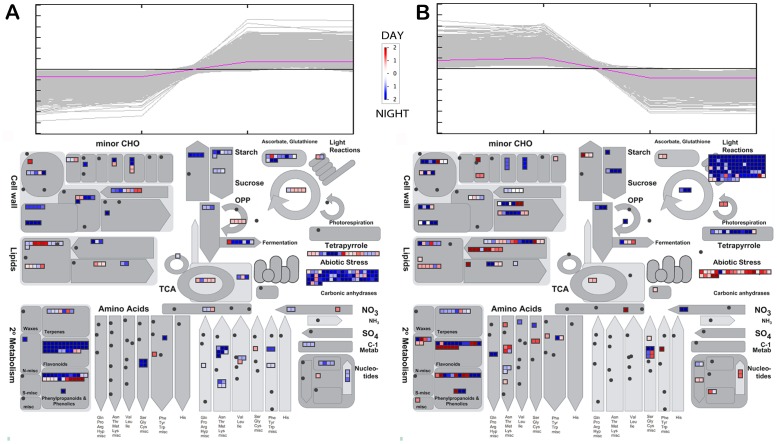
Example of genes allocated to illustrated clusters (4A: cluster 1 and 4B: cluster 2) specifically during day (red) or night (blue). Scales are log_2_ values calculated between G2 and R1.

The previous 9273 developmentally regulated transcripts were allocated to the same number of clusters, treating day and night samples separately. Both independent hierarchical clusterings yielded very similar expression patterns for day and night (Figure 3), however, a large number of transcripts differed between day and night in corresponding clusters. Functional categories over-represented in each cluster were obtained through enrichment analysis (Figures S1 to S4). Transcripts induced during ripening (cluster 1) only during the day or at night are illustrated in Figure 4A together with those repressed during ripening (cluster 2; Figure 4 B). This highlights developmentally regulated processes and their diurnal dependence. Flavonoid metabolism, amino acid metabolism and cell wall-related processes were noticeably induced in ripening berries during the night and not specifically during the day. A large number of photosynthesis-related genes were repressed only at night between young and ripening stages. This highlights the need to include nighttime sampling in developmental studies in order to investigate a substantially wider range of transcriptomic changes.

### Day/Night Modulated Transcripts

**Figure 5:**
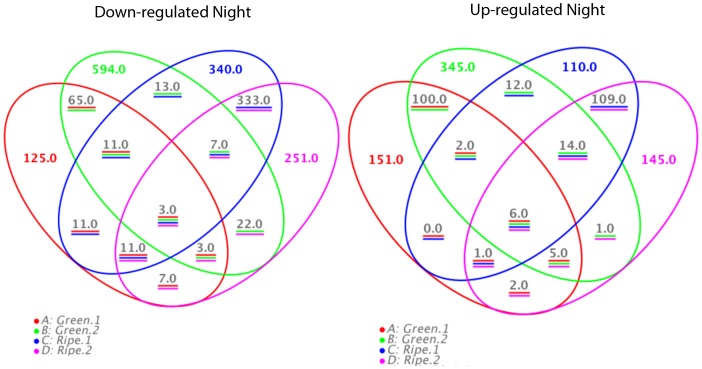
Overview of day/night modulated transcripts (fold change >2; pval adj <0.05) in each developmental stage. Left diagram night down-regulated transcripts; Right diagram night up-regulated transcripts.

**Figure 6:**
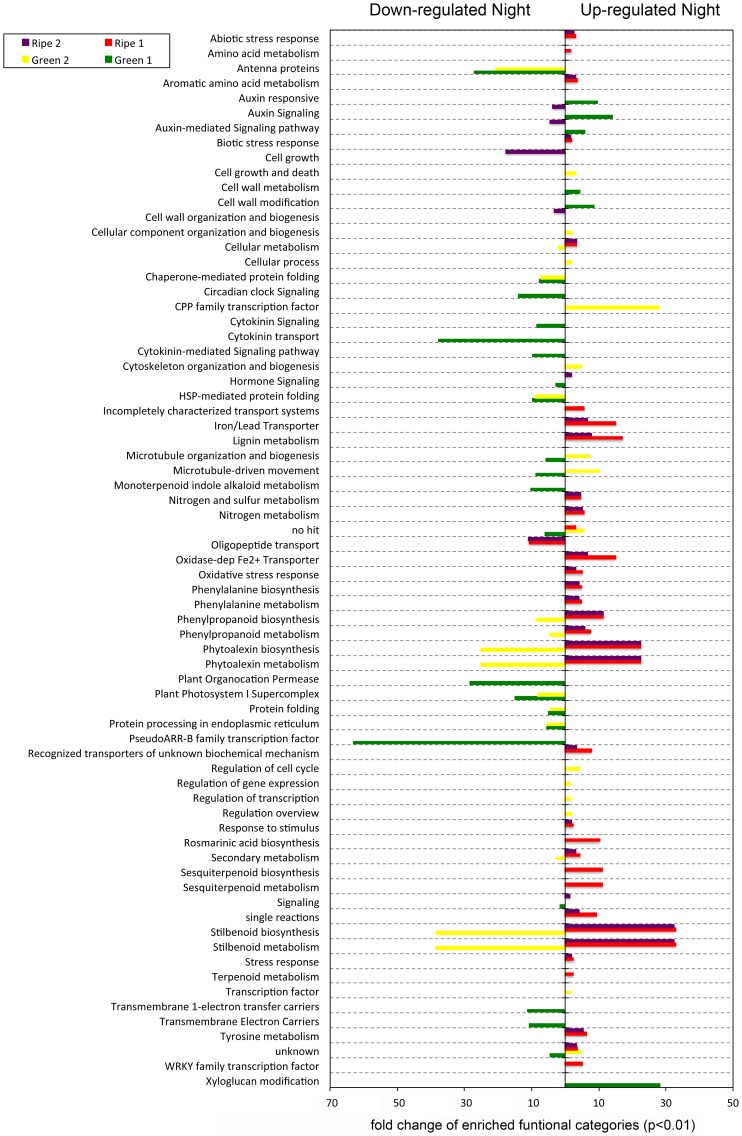
Fold change enrichment of functional categories (p<0.01) when compared to whole grapevine genome. Left part of the graph: night down-regulated transcripts and right part of graph night up-regulated transcripts at each analyzed developmental stage.

A second approach consisted of screening for genes differentially expressed between day and night at all four developmental stages (Figure 5). Surprisingly, very few transcripts (3 and 6) remained day/night modulated throughout berry development. This indicates that no pure mechanism of diurnal regulation prevails over all developmental stages. Many day/night-modulated genes were actually conserved within the green or the ripening group. In this respect, most genes in green berries were modulated between day and night in G2, whereas the differences in ripening berries were not as obvious. Berries at the end of the first growth period (G2) seem consequently to be most responsive to diurnal changes when compared to other stages. Functional classes of transcripts down- or up-regulated during the night were clearly separated between young and ripening berries (Figure 6). Modulated genes in young berries are mainly attributed to cellular division/expansion events that occur during the green growth phase (cell growth, cellulose catabolism, xyloglucan modification, microtubule-driven movement, oil entity organization). At green stages, the berry exhibits marked diurnal changes in volume consisting of night expansion followed by day contraction due to berry transpiration and water backflow to the canopy through xylem vessels [25], [49]. This large amplitude in cell expansion triggered at night places an additional demand on cell wall structural components. In ripening berries cell division has ceased and the diurnal pattern of swelling is strongly reduced by the impairment of xylem conductance preventing water backflow [25]. Consequently cellular growth-related categories are no longer significantly enriched within day/night- modulated transcripts. Photosynthesis (PS)- associated transcripts are repressed at night in the green berry, which may be due to the lack of light reactions of the PS system. In the ripening berry, diurnal changes of gene expression occur mainly within secondary metabolism, whereas categories like phenylpropanoid, terpenoid and stilbene biosynthesis were enriched in night-induced transcripts. Interestingly, genes within the latter category inverse their diurnal pattern between green and ripening berries. A switch from symplastic to apoplastic phloem unloading is known to occur in ripening berries [45], with hexoses (mainly fructose and glucose) being stocked in the vacuoles. Once ripening has started the berry has thus its own sugar reserves, which can be used for the synthesis of secondary metabolites.

### Indications of Oxidative Burst Occurring at Night in Ripening Berries

Oxidative burst is known to occur during ripening of climacteric fruit, but some studies have indicated that this phenomenon can also take place in non-climacteric fruit such as the grapevine [13], [46], [47]. Overexpression of genes involved in ROS scavenging peaking immediately after the onset of ripening was observed by several authors [17], [48], but its regulation at the transcriptional level remains unclear since these stress markers seemed to be absent in other studies [12]. Remarkably and what has never been previously observed, is that oxidative stress seems to occur in ripening berries at night, where functional categories related to oxidative stress response were enriched in up-regulated transcripts (Figure 6). This observation is confirmed by the fact that genes of the RBOH (respiratory burst oxidase protein) family *(VIT_14s0060g02320, VIT_01s0150g00440 and VIT_02s0025g00510*) are concomitantly induced at night in ripening berries (Table S4). RBOHs encode the key enzymatic subunit of plant NADPH oxidase and support the production of ROI (reactive oxygen intermediates) following biotic and abiotic stresses in plants [49]. Ascorbate oxidase isogenes (*VIT_07s0031g01040, VIT_07s0031g01120, VIT_07s0031g01120*) were also induced at night in R2 (Table S4). This family of ROI scavenging enzymes has been associated with the control of cell growth and the stress response [50]. A large number of peroxidase and laccase coding transcripts were found to be up-regulated in ripening berries at night (Table S4) in agreement with the night stress hypothesis. Ectopic expression of laccase in yeast confers improved H_2_O_2_ scavenging activity and hereby protect cells from lipid oxidative damage upon stress [51]. An up-regulation of RBOH could also be attributed to cell elongation at night during ripening. Studies on *Arabidopsis thaliana* RBOHc (*Atrbohc*) mutants indicated that ROIs activate hyper-polarization Ca^2+^ channels which are responsible for localized cell expansion during root-hair formation [52]. The induction of a calcium-transporting ATPase coding transcript (*VIT_13s0158g00360*) concomitant with calmodulin-binding proteins, and a calcium/proton exchanger (*CAX 3; VIT_08s0007g02240*; Table S4) may indicate day/night changes in the homeostasis of cytosolic Ca^2+^ in ripening berries. A cessation of Ca^2+^ importation actually results from the marked shift from xylem to phloem conductance at the onset of ripening [53]. In plants, stress initiates a signal-transduction pathway, in which the synthesis of γ-aminobutyric acid is increased [54]. This amino acid transiently accumulates in anoxic ripe berries and is rapidly re-oxidized upon restitution of air supply [55]. The up-regulation at night of a γ-aminobutyric acid transporter (*VIT_13s0074g00570*; Table S4) suggests that glutamate decarboxylase [56] and GABA shunt activities may be day/night modulated by changes in cytosolic Ca^2+^ (see above), pH, or redox state [57] in ripening berries. Furthermore, the transcription factor (TF) family WRKY was over-represented in R1 at night (Figure 6). TFs of the latter family were shown to respond to various types of biotic stress in rice [58].

Since growth in the ripening berry is due only to cellular expansion, the data suggests that this occurs mainly during the night. Additionally it supports the hypothesis presented in following section on carbohydrates that sugar importation into the ripening berry may principally occur during the night. It may also be hypothesized that sugar uploading into the vacuole increases osmotic pressure and thus represents a stressor for the cells.

### Carbohydrate Transport Related Transcripts Inverse their Day/Night Modulation from Green to Ripe Berries

Matching the pattern of sugar accumulation, sugar transporters (*ST; VIT_14s0006g03290, VIT_14s0083g00010, VIT_14s0083g00020, VIT_14s0083g00030, VIT_05s0020g03140*) were up-regulated in ripening berries (cluster 1 day and night; Table S3 and S5) concomitantly with hexose transporters (*HT1 and HT7; VIT_16s0013g01950, VIT_11s0149g00050*; Table S4). Curiously, all detected ST transcripts showed night up-regulation in the R2 (Table S4). This suggests that the apoplasmic pathway of sugar loading may be activated during the night with starch accumulation in chloroplasts occurring during the day and subsequent translocation as phloem-mobile sucrose during the night.

Interestingly *sucrose synthase* transcripts (*SuSy; VIT_05s0077g01930, VIT_10s0071g00070, VIT_00s1562g00010, VIT_11s0065g01130, VIT_12s0057g00130*) were induced during the night in green berries before the lag phase (Table S4). Frequently associated with sink tissues, *SuSy* are thought to be cytoplasmic enzymes in plant cells where they serve to degrade or synthesize sucrose and provide carbon for respiration and UDP-glucose for the synthesis of cell wall polysaccharides and starch [59], [60], [61]. It has also been reported that *SuSy* are tightly associated with the plasma membrane and therefore might serve to channel carbon directly from sucrose to cellulose and/or callose synthases in the plasma membrane [62]. This indicates that assimilated sugar is processed to cell wall compounds important for cell development in the night in green berries. Presented hypothesis is discussed in more detail in the section regarding cell division.

### Principal Events in the Phenylpropanoid Pathway Seem to be Regulated at Night during Ripening

**Figure 7:**
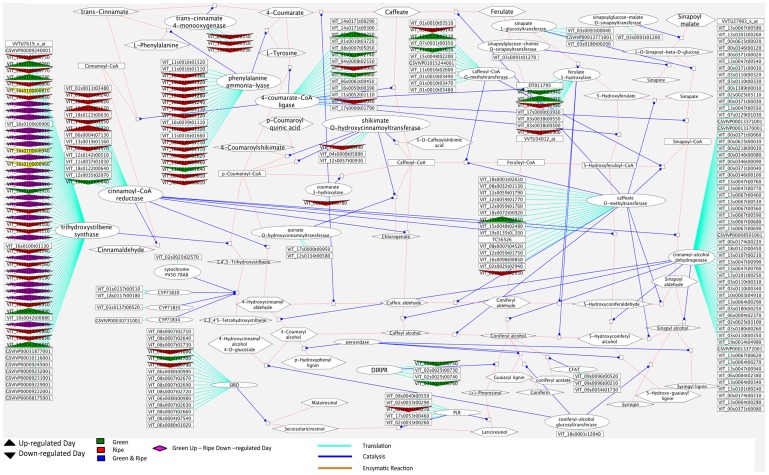
Cytoscape image of day/night modulated transcripts within the phenylpropanoid pathway. Only transcripts that were modulated at either, both green or both ripe stages (fold change >2; pval adj <0.05) are displayed. Arrow pointing upwards: up-regulated during the day (down-regulated at night); Arrow pointing downwards: down-regulated during the day (up-regulated at night). Green Arrow: Same regulation at G1 and G2; Red Arrow: Same regulation at R1 and R2; Purple Parallelogram: Day up-regulated in green stages; Day down-regulated in ripe stages; Magenta lines: Translation; Blue lines: Catalysis; Brown line: enzymatic reaction.

Phenolic compounds are important substances determining wine quality; they are partly responsible for color and astringency, and at the same time for numerous physiological benefits associated with moderate wine consumption [63]. Most phenolics derive from the non-oxidative deamination of phenylalanine via *phenylalanine-ammonia-lyase* (*PAL*) and encompass a range of structural classes such as lignins, phenolic acids, flavonoids and stilbenes [64]. Significant parts of the phenylpropanoid pathway and the day/night modulation of its enzymes are illustrated in Figure 7. A large number of isogenes within this pathway were repressed during the day (in relation to up-regulated at night) specifically at the ripe stages. In particular, almost all transcripts coding for the key enzyme PAL were up-regulated at night in ripe berries, signifying that major secondary processes take place during this final phase of development. Accordingly, transcripts coding the enzymes hydroxycinnamoyl-CoA shikimate/quinate hydroxycinnamoyltransferase (*VIT_11s0037g00440*) *and p-coumaroyl shikimate 3'-hydroxylase* (*VIT_08s0040g00780*), important elements of the shikimic acid pathway, were concomitantly modulated at night in ripening berries (Table S4). The shikimic acid pathway converts simple carbohydrate precursors derived from glycolysis and the pentose phosphate pathway to the aromatic amino acids tyrosine and phenylalanine, and thus provides the latter for the phenylpropanoid pathway [65]. Most transcripts coding for tri-hydroxy-stilbene-synthase, inversed their day/night modulation between the green and ripening stages (Figure 7) - they exhibited induction during night in ripening berries and vice versa in green berries. This implies that stilbene synthesis in ripening berries takes place during the night and vice versa during green growth stages, which is supported by the fact that *resveratrol synthases* (*RS*; *VIT_16s0100g01110, VIT_16s0100g01070*) are concomitantly regulated. *RS* intervenes in the final synthetic step of resveratrol, an important phytoalexin that has been shown to possess antioxidant and anti-inflammatory properties [66], [67].

Proanthocyanidin (PA) biosynthesis is part of the phenylpropanoid pathway that also produces anthocyanins and flavonols. PAs are polymers of flavan-3-ol subunits and often referred to as condensed tannins. They protect plants against herbivores, are important quality components of many fruits and constitute the majority of wine phenolics [68]. Two enzymes, *leucoanthocyanidin reductase* (*LAR*) and *anthocyanidin reductase* [69] can produce the flavan-3-ol monomers required for formation of PA polymers [70], [71]. Transcripts coding for *ANR* (*VIT_00s0361g00040*) and *LAR* (*VIT_17s0000g04150, VIT_01s0011g02960*) were consistently down-regulated throughout berry development (cluster 7; Table S5). The expression of the second *LAR* transcript in young green berries was twice as pronounced during the night as during the day throughout development (Table S3), underlining the importance of studying gene expression profiles at night. The induction of these enzymes in green berries concurs with current understanding that PA accumulation takes place in the early stages of berry development [71], [72]. Interestingly, *ANR* and *LAR* transcripts (*VIT_00s0361g00040, VIT_17s0000g04150*) were still up-regulated during the first ripening stage (R1) at nighttime together with the transcription factor *VvMYBPA1* (*VIT_15s0046g00170*), which regulates PA synthesis [77] (Table S3). Since no further PA synthesis is thought to take place during ripening, these results suggest that catechin and epicatechin monomers could accumulate in the night, while polymerization in tannosomes [73] would be blocked. Most of the secondary metabolites synthesized by plants are glycosylated, Williams and Harborne 1994 [74] characterized more than 1500 glycosides of flavonoids. Ford and Hoy, 1998 identified several classes of glycosylated secondary metabolites in grapevine berries, such as phenylpropanoids, including flavonols, anthocyanidins, flavanones, flavones, isoflavones, and stilbenes [75]. In this study isogenes of *UDP-glycosyltransferases* (*VIT_18s0001g06060, VIT_00s0324g00060, VIT_15s0046g01980, VIT_00s1251g00010, VIT_00s0324g00050*; Table S4) were induced during the night in R1, which coincides with the observations above of increased secondary metabolism. These diurnal expression profiles could partly explain the empirical observation that night cool temperatures are essential for the berry quality, which is partially linked to increased contents of secondary metabolites in grape berry skins [84].

Anthocyanin pigments are exclusively synthesized in berry skins during ripening [76]. Expression profiles of the principal genes involved in anthocyanin biosynthesis such as *UFGT* (*UDPglucose: flavonol 3-O-glucosyltransferase; VIT_04s0044g01540*), *VvMYBA1* (*VIT_02s0033g00380, VIT_02s0033g00410, VIT_02s0033g00440*) and *VvMYBA3* (*VIT_02s0033g00450*) were highly induced in ripening berries (cluster 1 day and night; Table S3 and S5) and thereby validate previous results obtained during day sampling on other *Vitis Vinifera* varieties [75], [81], [82], [83].

### Cell Division Events Occur to a Large Extent at Night in the Green Berry

The increase in volume and weight observed in grapevine berries during the first growth phase is due to cell division and expansion [4], [77]. During both early development stages, up-regulation of functional categories linked to cellular development was observed both day and night (cell growth and death, microtubule-driven movement, oil body organization and biogenesis; Figure 6). These transcriptomic changes are concomitant with the large increase in the quantity of cell DNA observed during the green growth stage [4]. Other authors have shown as well that cell wall biosynthesis and cytoskeleton organization take place during this phase, and that the related transcripts are subsequently down-regulated in ripening berries where no major changes in the composition of cell wall polysaccharide occurs [21], [78], [79].

All these categories showed noticeable diurnal variation in green berries. The xyloglucan functional category was highly over-represented in transcripts induced at night in G1 (Figure 6). Several transcripts coding for *xyloglucan endotransglycosylases* (*XET*; *VIT_11s0052g01200, VIT_11s0052g01180, VIT_11s0052g01280, VIT_01s0026g00200, VIT_11s0052g01270, VIT_11s0052g01300*) were also induced at night in G1 (Table S4). Xyloglucan (XG) is a primary cell wall hemicellulose that coats and cross-links cellulose microfibrils. XETs can cut and rejoin XG chains, and are therefore considered a key agent regulating cell wall expansion and are believed to be the enzyme responsible for the incorporation of newly synthesized XG into the wall matrix [80]. The expression pattern of these enzymes implies an activation of cell wall biosynthesis during the night in green berries. Several other profiles of transcripts involved in cell wall related processes point in the same direction. Cell division cycle protein 45 (*CDC45*; *VIT_12s0142g00280*), which interacts in the MCM (mini-chromosome maintenance) complex and plays a central role in the regulation and elongation stages of eukaryotic chromosomal DNA replication [81], [82] was night induced in G2. In addition *CDC7* (*VIT_15s0021g01380, VIT_00s0616g00030*), which triggers a chain reaction resulting in the phosphorylation of the MCM complex and ultimately in the initiation of DNA synthesis [83] were concomitantly modulated with CENP-E-like kinetochore proteins (*VIT_13s0067g03250, VIT_13s0067g03230*), a centromere protein (*VIT_00s0313g00010*) and a putative cell elongation protein (*VIT_01s0010g01200*; Table S4). Kinetochores are needed at the onset of mitosis, where cells break down their nuclear envelope, form a bipolar spindle and attach the chromosomes to microtubules [84]. Indications of enhanced cell division are also given by an up-regulation at night in G2 (Table S3) of DNA-binding proteins (*VIT_15s0048g00780, VIT_02s0025g05100*) and a DNA helicase (*VIT_16s0013g00300*). The transcript expression pattern observed here confirms literature data from a molecular point of view where cell multiplication occurs mostly in very young berries [4]. However, to the best of our knowledge, these results are the first on fleshy fruit demonstrating that important processes related to cell division preferentially occur during the night.

The microtubule-driven movement functional category mainly consists of members of the kinesin family. Kinesins are responsible for intracellular trafficking of vesicles and organelles along microtubules and for the dynamics of chromosomes and microtubules in mitosis and meiosis [85], [86]. These processes seem to occur mainly in more developed green berries (G2) (cluster 3; Figure S2). In addition transcripts within this category showed night up-regulation at G2 and curiously inversed their day/night modulation in young green berries (G1; Table S4). Recently, it has been proposed that kinesins intervene through transcriptional activation activity in regulating gibberellin biosynthesis and cell elongation [87]. This could explain the enrichment of this category in the more advanced green berries where cell division slows down and cell growth is more due to elongation. Since this category can only be observed during nighttime development, it is likely that this event has never been observed in prior transcriptomic studies in the grapevine.

### No Clear Evidence of a Pure Transcriptional Regulation of Malic Acid Metabolism was Observed

Malic acid accumulates very rapidly during the first growth phase and decreases throughout the second growth phase until harvest. The switch from malic acid net accumulation to degradation occurs at the onset of ripening [6], [88], [89]. Synthesis takes place in the cytosol, through carboxylation of phosphoenolpyruvate (PEP) provided from glycolysis, to oxaloacetate (OAA) by phosphoenolpyruvate carboxylase (PEPC) and further reduction into malate (MA) by cytosolic NAD-dependent malate dehydrogenase (NAD-MDH). Two transcripts coding for *PEPCs* (*VIT_01s0011g02740, VIT_12s0028g02180*) were repressed following the induction of ripening (cluster 2 day and cluster 7 night; Table S3 and S5). This regulation matches the developmental pattern of malate in berries. However, *PEPC* isogenes (*VIT_19s0015g00410, VIT_19s0015g00420, VIT_12s0028g02180*) were observed, exhibiting opposite expression patterns (cluster 1; Table S5). *NAD-MDH* transcripts (*VIT_10s0003g02500, VIT_03s0088g01190; VIT_15s0021g02410, VIT_10s0003g01000, VIT_10s0003g01000, VIT_01s0010g03090, VIT_19s0014g01640*; Table S5) also showed very variable patterns throughout development. These molecular data mirror the fact that berries can form malate from ^14^CO_2_ at any stage of development [90] and that enzymes involved in MA synthesis are not systematically down-regulated during ripening when no more net accumulation of MA occurs. This observation is in accordance with the literature where no relationship between MA content and the activities of PEPC or malic enzyme were observed in low and high acid peach cultivars [91], the acidless grape mutant *Gora Chirine* [92], apple [93], [94] and in low and high acidic loquat cultivars [95]. It therefore seems unlikely that MA accumulation is determined by the activity of these pathways. In plants, both the PEPC and malic enzyme (ME) are regulated by pH in a way that contributes to the stabilization of cytoplasm pH [25], [96], [97], [98].

The reactions involved in malic acid breakdown are oxidation by the Krebs cycle, gluconeogenesis, fermentation reactions that produce ethanol, anthocyanin synthesis, and amino acid inter-conversions [88], [99], [100]. Degradation takes place both in the cytosol, by oxidation into pyruvate and PEP via malic enzyme (ME) and phosphoenol-pyruvate-carboxykinase (PEPCK), respectively, and in the mitochondria, where MA is a substrate for the citrate cycle [101]. It should be noted that mitochondria purified from ripening berries cannot oxidize malate in the absence of added pyruvate, exactly as if the plant-specific mitochondrial ME was lacking [117]. Ruffner *et al*. (1976) [102] reported an increase in PEPCK activity in ripening grapes which coincides with two PEPCK transcripts found by Terrier et al. (2005) [12]. In microvine berries two *PEPCKs* were consistently up-regulated throughout development (*VIT_00s2840g00010, VIT_07s0205g00070*; cluster 8; Table S5). Together with the observed up-regulation of *MDHs* (*VIT_15s0021g02410, VIT_10s0003g01000, VIT_10s0003g01000, VIT_01s0010g03090, VIT_19s0014g01640*) these results confirm that the neoglucogenic pathway via OAA (catalyzed by MDH) and PEP (catalyzed by PEPCK) is active in the ripening berry. Functional studies on purified membrane vesicles clearly suggest that malate metabolism is controlled by changes affecting the bioenergetics of energy coupling at the vacuolar membrane in fruits [89]. In *Arabidopsis thaliana*, malate vacuolar transport is mediated by tonoplast dicarboxylate transporters (TDTs) [103] and members of the aluminum-activated malate transporter family (ALMT) [104]. *AtALMT9* and *AtALMT6* channels were associated with low fruit acidity in apples [105]. In the present study, *ALMT1* isogenes were detected (*VIT_08s0105g00250, VIT_09s0018g00890, VIT_06s0009g00450, VIT_06s0009g00480*; Table S5) and allocated to different clusters during the day and at night, but showed a tendency to down-regulation during berry development. Curiously, two of these isogenes (*VIT_06s0009g00450, VIT_06s0009g00480*) were significantly down-regulated between G2 and R1 at night (Table S3), whereas the others did not show any changes between two consecutive stages. ALMT1 seems hence not to trigger MA breakdown. By contrast, *ALMT9* isoenzymes (*VIT_02s0025g00700, VIT_18s0122g00020*) were induced from G2 to R1 (Table S3). This suggests possible involvement of *ALMT9* in MA metabolism transporting it from the vacuole to the cytoplasm to be catabolized by MDH and PEPCK.

### Tartaric Acid Regulation Does not Show Significant Day/Night Variation

Tartaric acid (TA) is quantitatively the most important acid in the mature berry [106]; as it is not used in primary metabolic pathways after the onset of ripening, the drop in tartaric acid concentration during ripening is due to dilution from water import, whereas the amount of tartaric acid per berry remains fairly constant [6], [107], [108]. As it is not directly affected by climatic conditions, TA is a very important wine quality-determining compound, in particular in warm climatic regions, and in the context of climate warming where malic acid is consumed rapidly resulting in a drop in total acidity and an increase in wine pH. TA synthesis occurs in the early stages of berry development immediately after fruit set and it levels off before the lag phase [5]. Ascorbic acid (Asc) has been proposed as its precursor with *L-idonate dehydrogenase* (*L-IdnDH*) showing its highest expression in young green berries as the main rate-limiting enzyme in the TA synthesis pathway [109]. *L-IdnDH* (*VIT_16s0100g00290*) was down-regulated throughout berry development (cluster 7; Table S5), matching the pattern of TA synthesis. Specific modulation at any of the green stages was not observed which is to be expected because *L-idhDH* transcripts are most abundant when TA synthesis starts in the very early stages of development. The down-regulation from G2 to R1 was twice as great during daytime development as during the night. In addition *L-IdhDH* night up-regulation was observed in the ripening berry without any apparent physiological reason (Table S4).

Asc as the major precursor of tartaric acid is synthesized by the Smirnoff-Wheeler pathway from L-galactono-1,4-lactone produced from GDP-L-mannose by the sequential action of *GDP-mannose-3,5-epimerase* (*GME*), *GDP-L-galactose phosphorylase* (*VTC2*), *L-galactose-1-phosphate phosphatase* and *L-galactose dehydrogenase* (*L-GalDH*), the direct ascorbate precursor [110]. Galacturonic acid from cell walls was suggested as an alternative major precursor of ascorbate with *galacturonate reductase* as a possible regulatory step enzyme [111]. Three *VTC2* isoenzymes were detected of which two (*VIT_14s0006g01370, VIT_10s0003g05000*) were slightly up-regulated throughout berry development (cluster 1 and cluster 7; Table S5). Only one (*VIT_19s0090g01000*; cluster 2) was down-regulated as expected given its putative role in TA synthesis, which ceases just before the lag phase.

### Day Seems to be as Important as Night in Amino Acid Metabolism

Free amino acids and ammonia make up the majority of nitrogen (N) containing compounds. Half of the berry’s total nitrogen is imported during ripening where proline (pro) and arginine (arg) account for over 70%. Only α-amino acids (pro is not fermented) are important yeast nutrients and thus needed for successful alcoholic fermentation [112], [113]. In addition they contribute to a considerable extent to varietal flavor in the finished wine [114].

In this study, most analyzed amino acids exhibited a steady increase from fruit set throughout ripening (Table S1.) Only glutamine (gln) was accumulated very early and steadily from berry set (BS) to G2 and thereafter decreased slightly from R1 to R2. Gln is a nitrogen donor for many biosynthetic reactions, including the biosynthesis of other amino acids, purines, pyrimidines, glucosamime and carbamoyl phosphate and its biosynthesis is catalyzed by glutamine synthetase. Consistently *glutamine synthetase* isogenes (*VIT_16s0100g00580, VIT_03s0088g00570, VIT_05s0020g02480*; Table S3) were highly up-regulated at G2 and three other isogenes were induced from young to ripening stages (*VIT_07s0104g00170, VIT_08s0007g04670, VIT_10s0042g01000*; Table S5).

A transcript coding for *NADH glutamate synthase* (*VIT_07s0005g00530*) which catalyzes the reaction from gln to glutamate (glu) was down-regulated (cluster 2, day and night) in ripening berries in addition to *GLT1* (*NADH-dependent glutamate synthase 1*) genes (*VIT_16s0098g00290, VIT_15s0024g01030*), where the second transcript was only detected during daytime development. The complex regulation of glu and gln does not permit any conclusive statement to be made about the molecular events occurring during berry development during the day and at night.

In grapevine berries, pro accumulation starts very late during the first growth phase and continues throughout ripening [115], arg, the other principal amino acid, which shares significant pathway features with pro, begins to accumulate earlier in the green berry and continues during ripening. Arg accumulation levels off early during ripening in cultivars exhibiting very high pro concentrations [116], which, on the basis of this study, also seems to be true for the microvine. Arg was present in green berries, but a significant increase was observed both in pro and arg, in particular in ripening berries. There are two pathways of pro biosynthesis in higher plants. The first is from glu, which is converted to pro by two successive reductions catalyzed by *pyrroline-5-carboxylate synthase* (*P5CS*) and *pyrroline-5-carboxylate reductase* (*P5CR*), respectively. *P5CS* is a bifunctional enzyme catalyzing firstly the activation of glu by phosphorylation and secondly the reduction of the labile intermediate c-glutamyl phosphate into glutamate-semialdehyde (GSA), which is in equilibrium with the P5C form [117], [118]. Although it has been shown that pro accumulation in grapes occurred independently from *P5CS* which was expressed evenly during berry development and in which other regulation systems probably intervene [115], we detected *P5CS* isogenes (*VIT_15s0024g00720, VIT_08s0007g01060*), which were up-regulated in ripening berries (cluster 1; Table S5) where pro is accumulated. This is in agreement with other microarray studies carried out on Cabernet Sauvignon [18] and Trincadeira [17]. Moreover, three pro transporter isogenes were detected (*VIT_13s0019g03220, VIT_13s0073g00290, VIT_07s0141g00640*; Table S3) and correlated with pro accumulation during up-regulation from G2 to R1 without showing any day/night specificities.

An alternative pathway starts with the pro precursor ornithine, which can be transaminated to *P5C* by ornithine aminotransferase (OAT), a mitochondrial-located enzyme [119]. An OAT transcript (*VIT_10s0003g03870*) was down-regulated in G1 (cluster 6; Table S5) during the day, suggesting that this pathway may not be important in green berries. A *glutamate decarboxylase* (GDC) transcript (*VIT_01s0011g06610*) producing γ-aminobutyrate was induced in green berries before the lag phase, and then continuously down-regulated (cluster 7 day and night; Table S5). The latter transcript exhibited as well a day induction in G2. As γ-aminobutyrate is also a stress marker this could explain the daytime up-regulation in response to higher day temperatures in green berries.

Lysine-histidine transporters (LHT) show a very high affinity for amino acids, and LHT1 in particular belongs to a class of amino acid transporters that is specific for lys and his [120]. It has been shown that *LHT1* is involved in the uptake of amino acids from soil into the leaf mesophyll cells [121]. No clear pattern in *LHT1* isogenes was observed in this study: Some isogenes (*VIT_01s0010g02500, VIT_01s0010g02510, VIT_01s0010g02520*) were up-regulated in G1 (cluster 5 day and night; Table S5) whereas others (*VIT_06s0061g01210, VIT_14s0171g00400*, cluster 8 day and 9 night; Table S5) showed opposite patterns.

### Genes Involved in Terpene and Carotenoid Biosynthesis Show Circadian Patterns

Terpenoid volatiles, principally monoterpene alcohols such as linalool, geraniol, nerol and terpineol are important flavor and aroma compounds of grapevine berries and wine, and most accumulate during ripening [122], [123]. For example, in fruits of the cultivar Muscat, the terpenoid content paralleled sugar accumulation and several monoterpenes reached peak levels in the overripe fruit [124], though present molecular data does not unambiguously confirm this. Monoterpenes are products of the isoprenoid pathway from the intermediates isopentenyl-pyrophosphate (IPP) and its isomer dimethylallyl pyrophosphate (DMAPP). IPP is synthesized via the non-mevalonate pathway that requires *1-deoxy-D-xylulose 5-phosphate synthase*. The transcript coding for this enzyme (*VIT_09s0002g02050*) was consistently down-regulated during berry development (cluster 7 day; cluster 2 night; Table S5) whereas *isopentenyl diphosphate isomerase 2* transcripts, catalyzing the conversion of IPP to DMAPP were induced in ripening berries (*VIT_00s0768g00030, VIT_04s0023g00600, VIT_11s0206g00020*, cluster 1 day and night; Table S3 and S5).

Geraniol 10-hydroxylase (G10H) is thought to play an important role in iridoid monoterpenoid and indole alkaloid biosynthesis [125]. Most *G10H* transcripts were induced in ripening berries to the same degree at day and night (cluster 1; Table S5). However, two transcripts (*VIT_02s0012g02370, VIT_02s0012g02380*) showed nighttime induction in ripening berries, which was most pronounced at the latest stage (Table S4). Several transcripts coding for the enzymes involved in the biosynthesis of the bicyclic monoterpene pinene were found to be modulated. Pinene has a woody-green pine aroma and is one of the most widely detected volatile organic compounds emitted by plant into the atmosphere [126]. Several homologues of *pinene synthase* showed down-regulation in ripening berries (Table S3 and S5). Two of the transcripts (*VIT_08s0007g06860, VIT_12s0059g02710*) were induced at night in R1 (Table S4). The tendency to exhibit a circadian expression pattern of pinene synthase-coding transcripts has been observed in *Artemisia annua* [126], but here this day/night pattern was observed at only one berry developmental stage.

Two sesquiterpene synthases, (+)-valencene- and (−)-germacrene D-synthase have been recently characterized in *Vitis Vinifera* L. berries. Their expression was principally induced during later stages of berry development, several weeks after the onset of ripening [127]. Consistent with this, it was found that a *valencene synthase* (*VIT_18s0001g04050*) and a *(*−*)-germacrene D synthase* were induced in ripening berries (Table S3). Several isogenes of *(*−*)-germacrene D synthase* exhibited night up-regulation in R1 (*VIT_18s0001g04550, VIT_18s0001g04120, VIT_18s0001g04780, VIT_18s0001g05240*; Table S4) suggesting a circadian regulation amongst genes in terpene biosynthesis.

An important subgroup of terpenes are carotenoids, a heterogeneous group of plant isoprenoids primarily present in the photosynthetic membranes of all plants where they quench triplet chlorophyll, singlet oxygen, and also superoxide anion radicals [128]. The first committed step in carotenoid biosynthesis is the production of the 40-carbon phytoene from condensation of two geranylgeranyl pyrophosphate (GGPP) molecules, catalyzed by the enzyme phytoene synthase (PSY). Three *PSY*s were detected showing opposite expressions hence not presenting a consistent pattern during berry development (Table S5).

The cleavage of carotenoids can lead to the formation of C_13_-norisoprenoids and the phytohormones abscisic acid and strigolactone. C_13_-norisoprenoids are important flavor compounds contributing to varietal character of grapes and wine. In the grapevine, a direct relationship between a decrease in carotenoid concentration and C_13-_norisoprenoid production has been demonstrated [129]. The C_13_-norisoprenoids identified in wine with important sensory properties are TCH (2,2,6-trimethylcyclohexanone), β-damascenone, β-ionone, vitispirane, actinidiol, TDN (1,1,6-trimethyl-1,2-dihydronaphthalene), riesling acetal and TPB (4-(2,3,6-trimethylphenyl)buta-1,3-diene) [130]. The principal enzyme involved in the cleavage of carotenoids to C_13_ norisoprenoids is carotenoid *cleavage dioxygenase 1* (*CCD1*), which has been characterized in grapes where it exhibited an induction of gene expression towards ripening [131]. In the present study a putative *CCD1* homologue (*VIT_02s0087g00930*) was identified that was highly up-regulated towards ripening (cluster 1 day and night; Table S3 and S5) supporting the results obtained by Mattieu *et al*., 2005 [131] where C_13_-norisprenoid synthesis takes place rather in ripening berries occurring after *CCD* induction.

### Circadian Clock Related Transcripts Followed Day/Night Patterns Mainly in Green Berries

The circadian clock consists of morning, core, and evening interlocking feedback loops [132]. The MYB transcription factors *CCA1* (*circadian clock associated1*) and *LHY* (*late elongated hypocotyl*) belong to the core loop in *Arabidopsis thaliana* [29]. *CCA1* regulates homeostasis of ROS (reactive oxygen species) and would thus coordinate time-dependent responses to oxidative stress [133]. In both green stages, a *CAA1* transcript (*VIT_15s0048g02410*; Table S4) was considerably induced at night while *LHY* responded only in G1. *CIR1*, a third circadian clock-related transcript putatively involved to the core loop (*VIT_04s0079g00410*; Table S4) was found to be day/night modulated at all stages but R2. The morning loop induces *PRR9* and *PRR7* (pseudo response regulator) that are linked to *CCA1/LHY* [134], [135]. In microvine berries isogenes of *PRR7* (*VIT_13s0067g03390, VIT_06s0004g03660, VIT_06s0004g03650*), *PRR9* (*VIT_15s0048g02540*) and a *PRR5* (*VIT_16s0098g00900*) were concomitantly induced during the day but only in the first green stage of berry development (Table S4). A putative *GI* (*gigantea*) transcript (*VIT_18s0157g00020*) identified in the evening loop [136] and epistatic to *ELF4* (*early flowering 4*) [137] was down-regulated at G1 whereas *ELF4* (*VIT_13s0067g00860*) showed night induction at both G1 and G2. A homologue (*VIT_07s0104g00350*) to ZGT acting as a coupling agent between the central circadian oscillator and rhythmic *LHCB1* (*light harvesting complex*) was induced during the day in G1 and G2. It may be concluded that green berries are significantly more responsive to the circadian cycle than ripe berries. It can be hypothesized that this is due to the fact that ripe berries have reserves in the form of fructose and glucose, whereas green berries photosynthesize during the day and many genes associated with the circadian clock are somehow involved in photosynthesis also.

### Heat Shock Related Genes and Transcription Factors Change their Day/Night Expression Pattern According to Developmental Stage

The *multi-protein-bridging factor 1c* (*MBF1c*) previously characterized in *Arabidopsis thaliana* functions upstream to salicylic acid, ethylene and trehalose upon heat stress [138], [139]. In microvine berries, *MBF1c* showed consistent up-regulation towards ripening (*VIT_11s0016g04080*; cluster 6 day and cluster 8 night; Table S5). This heat shock responsive transcription factor would be expected to be daytime induced as well due to the temperature gradient between day and night (ΔT_day_ +10°C). *MBF1c* was induced during the day in green berries, but no modulation was observed in ripe berries, indicating a higher temperature sensitivity of the green berry.

*VvGOLS1* (*galactinol synthase)* has recently been identified as being temperature regulated in berries of Cabernet Sauvignon L. [140]. This gene is transactivated by the heat shock transcription factor *VvHSFA2* [140]. In microvine berries, several *galactinol synthase* coding isogenes were modulated throughout berry development and/or during the day/night (Table S3–S5). Ten of these probe sets exhibited day/night co-regulation - all were day up-regulated in green berries and inversely modulated in ripening berries. However, they did not show a common pattern of regulation throughout berry development. The *VvGOLS1* gene locus (*VIT_07s0005g01970*) from Pillet et al., 2012 [141] showed consistent up-regulation throughout development (cluster 6 day and cluster 8 night) and day induction only at G2. As the day/night temperature gradient was +10°C it was expected that *VvGOLS1* would be activated during the day as it is very responsive to heat stress. However, in ripening berries, it seemed to loose this function like *MBF1c*: circadian changes appeared thus to have greater impact than the day/night temperature gradient.

In plants, bHLH (basic helix-loop-helix) proteins function as transcriptional regulators modulating secondary metabolism, fruit dehiscence, carpel and epidermal development, phytochrome signaling, and responses to environmental factors [141], [142], [143]. This functional category showed continuous down-regulation throughout berry development, with a peak in the young green berry where major events in early reproductive development occur (cluster 7, Figure S3). Furthermore, enrichment could be observed only during night development, confirming the previous hypothesis that significant changes in cellular division take place at night in green berries, as supported by the expression pattern of a transcript coding for SPATULA (*VIT_18s0001g10270*) which affected cell proliferation in *Arabidopsis thaliana* [144].

### Ethylene

As the grapevine fruit ripens without ethylene and does not exhibit a respiration burst nor high production of ethylene it has consequently been classified as non-climacteric [145]. However, Chervin *et al*., 2004 [146] reported a modest increase in ethylene at the onset of ripening in the grapevine. The same authors observed a correlation between ethylene accumulation and the expression of *1-aminocyclopropane-1-carboxylate oxidase* (*AOC*) transcripts and enzyme activity in berries. *AOC* catalyzes the final reaction step from ACC to ethylene [147] and has been also identified in the wall of apple and tomato fruit cells [148]. Eleven *AOC* isogenes were detected without exhibiting a common pattern throughout development. However ethylene receptor coding transcripts (*ETR1; VIT_19s0093g00580, ETR2; VIT_06s0004g05240*) were induced during development in ripening berries (*ETR1*: cluster 1 night, cluster 6 day and *ETR2*: cluster 1 night, cluster 8 day; Table S5). In addition to these developmental regulations, *ETR2* showed nighttime induction in ripening berries (Table S5). These results support the hypothesis of ethylene intervention in berry ripening whose role might be in relation to berry architecture or anthocyanin accumulation [146], [149]. Taking this into account, together with the observed abundance of principal phenylpropanoid pathway transcripts at night in ripe berries, putative involvement of ethylene in secondary metabolism could be supposed. However, indications do exist that the circadian rhythm plays a critical role in ethylene regulation and should be taken into account in further hormonal studies.

### Abscisic Acid

Abscisic acid (ABA) intervenes in embryo and endosperm formation during seed development, in seed dormancy in mature berries and has a promotive role during fruit ripening [98]. Highest ABA levels are found in very young berries, which then decrease until ripening, where accumulation resumes in parallel with coloration and sugar accumulation [145], [150]. The rate limiting enzyme in ABA synthesis, *9-cis-epoxycarotenoid dioxygenase* [151] (*NCED; VIT_02s0087g00930*), steadily increased throughout berry development (cluster 5; Table S5), which is in agreement with previous studies on other varieties [18]. Another important enzyme involved in ABA synthesis is *zeaxanthin epoxidase* (*ZEP*), which catalyzes zeaxanthine biosynthesis, a carotenoid precursor for ABA [152]. There are few data on *ZEP* available - Deluc, *et al*. 2007 [18] observed a steady decrease in expression in Cabernet Sauvignon L. during berry development, the same pattern of *ZEP* transcripts (*VIT_00s0533g00020; VIT_13s0156g00350, VIT_07s0031g00620*; cluster 2 night, cluster 7 day; Table S5) was found in microvine berries at day and night.

An *NCED* transcript was found to be induced during the day in green berries but this expression was inversed in R1 (*VIT_19s0093g00550*; Table S4). In *Arabidopsis thaliana* induction of this enzyme led to greater stress tolerance to intense light and high temperatures [153]. *CYP707A1* (*VIT_02s0087g00710*) and *CYP707A2* (*VIT_07s0031g00690*) encode for *abscisic acid 8'-hydroxylases* which controls seed dormancy and germination in *Arabidopsis thaliana* [154]. Interestingly, they also exhibited nighttime up-regulation of *CYP707A1* at all stages but in young green berries *CYP707A2* was induced only in G2 (Table S4). Generally ABA also plays a role in abiotic and biotic stress tolerance in plants [155], thus these results reinforce the observation that oxidative stress appears to occur during the night in ripening berries. However, the opposite was observed in regards to the ABA-mediated signaling category, which was significantly enriched in transcripts down-regulated at night in R1 (Figure 6). This was mainly due to isogenes of *ATHVA22A* (*Arabidopsis thaliana HVA22 homologue A*) that were up-regulated during the day in R1 and in R2 (Table S4). *HVA22* is mediated by ABA and was induced by cold and drought stress in barley [156]. It has been shown that HVA22 is a ER- and golgi-localized protein that negatively regulates GA-mediated vacuolation and programmed cell death [157]. This regulation pattern cannot be explained by temperature neither by the previously described oxidative stress hypothesis occurring at night in ripening berries. Nonetheless, it shows though that the genes of this family appear to be moderately responsive to diurnal and developmental changes.

### Gibberellins

Gibberellins (GAs) are regulators of many plant development processes, mainly cell division and expansion. During the reproductive development of the grapevine, GAs are known to be involved in the regulation of grapevine fruit set and young berry expansion. Accordingly, GA levels during berry development are high around flowering and early in berry development and decrease steadily thereafter [158]. Two gibberellin receptor coding transcripts (*GID1L3; VIT_15s0048g01390, VIT_15s0048g01350*) were night up-regulated in R2 (Table S4). Similar night induction in ripening berries was observed in relation to Gibberellin oxidases (*GA 20ox2: VIT_03s0063g01290, VIT_03s0063g01280 and GA 2ox: VIT_05s0077g00520*), enzymes involved in GA metabolism in higher plants [159]. During berry development many isogenes coding for the above enzymes where allocated to different clusters exhibiting no clear expression pattern (Table S5). No conclusions can be drawn regarding GA developmental regulation; day/night expression patterns of detected transcripts indicate their putative involvement in secondary metabolism, which was found to be highly active at night in ripening berries.

### Cytokinins

Cytokinins intervene in the establishment of the vasculature during embryonic development; they control the number of early cell divisions and have a regulatory control on meristem activity and organ growth during postembryonic development [160]. In the grapevine berry they are thought to be involved in fruit set and growth promotion with maximum concentrations in young berries, decreasing towards ripening. [161]. Induction of transcripts was observed in young green berries, which are involved in mediating cytokinin reception and transport, such as *histidine kinase* (*AHK4/WOL; VIT_01s0011g06190*) acting as a cytokinin receptor protein [162], (cluster 5 day; Table S5). *Purine permease 1* (*PUP1; VIT_18s0001g06950, VIT_18s0001g06940, VIT_18s0001g06910*), involved in cytokinin transport [163] showed consistent up-regulation throughout berry development (cluster 6 day and cluster 8 night). Isopentenyltransferase, catalyzing the rate-limiting step in cytokinin biosynthesis in *Arabidopsis thaliana* [164] (*VIT_09s0070g00710, VIT_07s0104g00270*) was concomitantly regulated (cluster 6 day and night; Table S5) and exhibited additional up-regulation during the day in R1 (Table S4). It was not possible to confirm the results of Deluc *et al*., 2011 [18] who observed a steady decrease in a putative *cytokinin oxidase* during berry development, probably related to decreases in cytokinin content. In microvine berries three transcripts coding for a putative *cytokinin oxidase* (VIT_00s2520g00010, VIT_00s2191g00010, VIT_00s0252g00040) were strongly up-regulated (cluster 1; Table S5) in ripening berries, indicating that this enzyme probably does not play a major role in cytokinin synthesis. Many cytokinin-mediated transcripts were down-regulated at night in G1 (see functional category cytokinin-mediated signaling in Figure 6). Most of these probesets were homologues to the *pseudo-regulators* (*PRRs*) that were discussed above in the circadian clock section.

### Conclusion

To our knowledge this is the first genome-wide transcriptomic study on fleshy fruits deciphering night regulations throughout development, and comparing day/night gene expression changes at different stages. All developmentally regulated transcripts detected during the day were also detected at night, validating previous approaches based solely on day sampling. Day expression data was well correlated with other expression data obtained on a non-dwarf genotype grown in the field.

Here, advantage has been taken of the microvine model to perform simultaneous sampling of fruits at several developmental stages from the same plant. Due to the size of the microvine, experiments could be performed in climatic chambers under strictly controlled environmental conditions (i.e. day/night radiation, temperature, vapor pressure deficit) unprecedented in other development studies on grapevine fruit development. Thereby experimental noise, affecting gene expression in a non-quantifiable way, was reduced to a minimum. It was demonstrated that 20% of developmentally-regulated transcripts were only detected during the night and that very few transcripts are day/night regulated consistently throughout all stages of development. This indicates that photoperiod regulation drastically changes at the onset of sugar storage in berries. In many pathways, it was observed that the gene expression pattern showed a day/night variation with changes in relation to sampling stage. This is particularly noticeable with respect to cell wall-related processes that are more active during night in the young fruit. Significant observations were made in relation to secondary metabolism-related enzymes that were only present in the ripening berry during the night. Several processes showed an inversion of their day/night regulation between green and ripe berries, such as sugar transport and phytoalexin synthesis, which were more pronounced during the day in green berries and vice versa in ripening berries. Interestingly, the oxidative burst transiently detected by several authors at the onset of ripening was observed to occur at nighttime in the ripening berry.

For a greater understanding of the mechanisms involved in the regulation of berry development, it appears to be essential to evaluate different processes and events both during the day and at night. Considering the significant diurnal changes observed during this study on plants grown under controlled conditions, it would also seem necessary to investigate the transcriptomic response to abiotic stresses and its day – night modulation at different stages of development.

## Materials and Methods

### Plant Material

One year old own-rooted microvines were grown in a greenhouse until a stable fructification was established. The reproductive system was normalized among all plants by removing organs up to flowering. Plants were further grown in climatic chambers (2 m^2^). One whole developmental cycle was undergone under fully controlled conditions (day/night temperature: 30/20°C, Photoperiod: 14 h, VPD: 1 kPa). Reproductive organs were sampled in biological triplicates two hours before the end of the day and the end of the night and were immediately frozen in liquid N_2_. 30 berries per replicate were crushed into liquid N_2_ and the obtained powder was used for biochemical analysis and RNA extraction.

### Organic Acid and Sugar Analysis

For organic acid, glucose and fructose approximately 0.1 g of powder was diluted five fold in deionized water and samples were frozen at −20°C. Prior to analysis diluted aliquots were defrosted and subsequently heated (60°C for 30 min). After cooling to ambient temperature, samples were homogenized and diluted with 4.375 µM acetate as an internal standard. To avoid potassium bitartrate precipitation, 1 mL sample was mixed with 0.18 g of Sigma Amberlite® IR-120 Plus (sodium form) and agitated in a rotary shaker for at least 10 hours before centrifugation (13000 rpm for 10 min). The supernatant was transferred into HPLC vials before injection on Aminex HPX®87H column eluted in isocratic conditions (0.05 mL.min^−1^, 60°C, H_2_SO_4_) [165]. Organic acids were detected at 210 nm with a waters 2487 dual absorbance detector®. A refractive index detector Kontron 475® was used to determine fructose and glucose concentration. Concentrations were calculated according to Eyegghe-Bickong et al. 2012 [166].

### Amino Acid Analysis

Primary amino acids were analyzed using a modified version of a previously reported method [167]. A Hewlett-Packard (Agilent Technologies Massy, France®) 1100 179 series HPLC instrument was used, with a G1321A fluorescence detector set at excitation and emission wavelengths of 330 nm and 440 nm, respectively. Separations were carried out on a 150 mm×3 mm Macherey Nagel Durabond® column 5 µm dp, protected by a 1 mm C18 SecurityGuard® cartridge supplied by Phenomenex (France). Mobile phase A consisted of 95% 0.05 M acetate buffer, pH 6.5 and 5% methanol:acetonitrile [1∶1] filtered under vacuum using a 0.22 µm nylon membrane. Mobile phase B consisted of methanol:acetonitrile [1∶1]. Separations were carried out at 40°C with a flow rate of 0.5 ml/min. As proline does not react with OPA, a new high-throughput spectrophotometric method was developed and validated for its analysis. Briefly, the method involves reacting the sample with ninhydrin in DMSO and formic acid at 100°C for 15 minutes to yield a salmon pink reaction product. Under these conditions, primary amino acids do not react with ninhydrin and thanks to the particular solvent composition, the extraction and centrifugation steps reported in similar methods are avoided.

### RNA Extraction

RNA extraction was carried out using an in-house extraction buffer containing 6 M guandine-hydrochloride, 0.15 M tri-sodium-citrate, 20 mM EDTA and 1.5% CTAB. Five volumes of room temperature extraction buffer supplemented with 1% MSH were added to 1 g of powder followed by immediate agitation. Cell debris was removed by centrifugation, and after chloroform treatment one volume isopropanol was added to precipitate RNA. Samples were kept at –20°C for at least two hours. RNA was precipitated by centrifugation washed with 75% ethanol and the pellet was suspended with RLC Buffer from the Quiagen rnaEasy® Kit previously supplemented with 1.5% CTAB. To reduce pectin and tannin residues an additional chloroform treatment was carried out. The succeeding washing steps and the DNAase treatment are performed as described in the kit. Absorbance was measured at 260 and 280 nm and the concentration of RNA was determined with a NanoDrop 2000c Spectrophotometer (Thermo Scientific®). The integrity of RNA was evaluated using an 2100 Bioanalyzer (Agilent Technolgies®).

### Nimblegen 12x Microarray Hybridization

cDNA synthesis, labeling, hybridization and washing reactions were performed according to the NimbleGen Arrays User's Guide (V 3.2). Hybridization was performed on a NimbleGen microarray 090818 Vitis exp HX12 (Roche, NimbleGen Inc., Madison, WI), consisting of 29,549 predicted genes on the basis of the 12X grapevine V1 gene prediction version V1 http://srs.ebi.ac.uk/. The chip probe design is available at the following url: http://ddlab.sci.univr.it/FunctionalGenomics/. The raw data is available at the Gene Expression Omnibus (http://www.ncbi.nlm.nih.gov/geo/info/linking.html) under the series entry GSE52829.

### Statistical Analysis

The Robust Multi-array Analysis (RMA) algorithm was used for background correction, normalization and expression levels [168]. Differential expression analysis was performed with the bayes t-statistics from the linear models for microarray data (limma) [169]. P-values were corrected for multiple-testing using the Benjamini-Hochberg’s method [170]. Transcripts were considered as significantly modulated when absolute change was >2 fold (log_2_ fold change >1) and adjusted p. value was <0.05 between two conditions. Gene clustering was performed on mean centered values of RMA normalized and log_2_ transformed expression data. This analysis was performed using the Multiple Experiment Viewer version 4.6.2® software package, and based on the k-means method using Pearson’s correlation distance calculated on the gene expression profiles. Gene annotation was derived from Grimplet et al., 2012 [171].

### Visualization of Grapevine Transcriptomics Data Using MapMan Software

Information from the Nimblegen microarray platform was integrated using MapMan software [172] as described for the Array Ready Oligo Set *Vitis Vinifera* (grape), V1.0 (Operon, Qiagen), and the Affymertix GeneChip® *Vitis Vinifera* Genome Array [64] (correspondence from Grimplet et al.; 2012 [172]. Mapman pathway analysis was performed with day and night-specific transcripts allocated to cluster 1 and 2, respectively. For identified genes, the fold change between G2 and R1 was calculated and mapped on the pathway “metabolism overview”. Day-specific values were mapped in red and night-specific ones in blue.

### Cytoscape Pathway Analysis

For the illustration of the phenylpropanoid pathway, transcripts that were significantly and concomitantly modulated (fc >2, p<0.05) in either both green or both ripe stages were mapped using VitisNet networks through cytoscape v 2.8.3 s [173].

### Functional Categories

Transcripts allocated to day - night development clusters or identified by statistical testing were analyzed with FatiGO [174] in order to identify significant enrichment of functional category. Categories were derived form [171] and Fisher’s exact test was carried out to compare genes list with non-redundant transcripts from the grapevine genome. Significant enrichment was considered in case of p value <0.01 and illustrated as fold change.

## Supporting Information

Figure S1 **Fold change of enriched functional categories of transcripts allocated to cluster 1 and 2.** Categories for all day and night as well as for day and night specific transcript within cluster is illustrated.
(PDF)

Figure S2 **Fold change of enriched functional categories of transcripts allocated to cluster 3 and 4.** Categories for all day and night as well as for day and night specific transcript within cluster is illustrated.
(PDF)

Figure S3 **Fold change of enriched functional categories of transcripts allocated to cluster 5 and 6.** Categories for all day and night as well as for day and night specific transcript within cluster is illustrated.
(PDF)

Figure S4 **Fold change of enriched functional categories of transcripts allocated to cluster 7 and 8.** Categories for all day and night as well as for day and night specific transcript within cluster is illustrated.
(PDF)

Figure S5 **Correlation between genes expression (log_2_) between green and ripening stages of Corvina L. (Fasoli** ***et al.,*** **2012) and microvine berries.**
(BMP)

Table S1 Amino acid content of sampled berries.
(XLSX)

Table S2 Overview of the number of up and down-regulated transcripts within all developmental stages.
(XLSX)

Table S3 All modulated transcripts between developmental stages.
(XLSX)

Table S4 Day – Night modulated transcripts.
(XLSX)

Table S5 Transcripts allocated to clusters.
(XLSX)

Table S6 Transcripts, identified in Corvina L. as well as in microvine berries between green and ripe stages.
(XLSX)
