# Supplementary figures and images for: Is Transcriptomic Regulation of Berry Development More Important at Night than During the Day?
Source: PLoS One. 2014 Feb 13;9(2):e88844. doi: 10.1371/journal.pone.0088844 (PMC3923830; doi:10.1371/journal.pone.0088844)

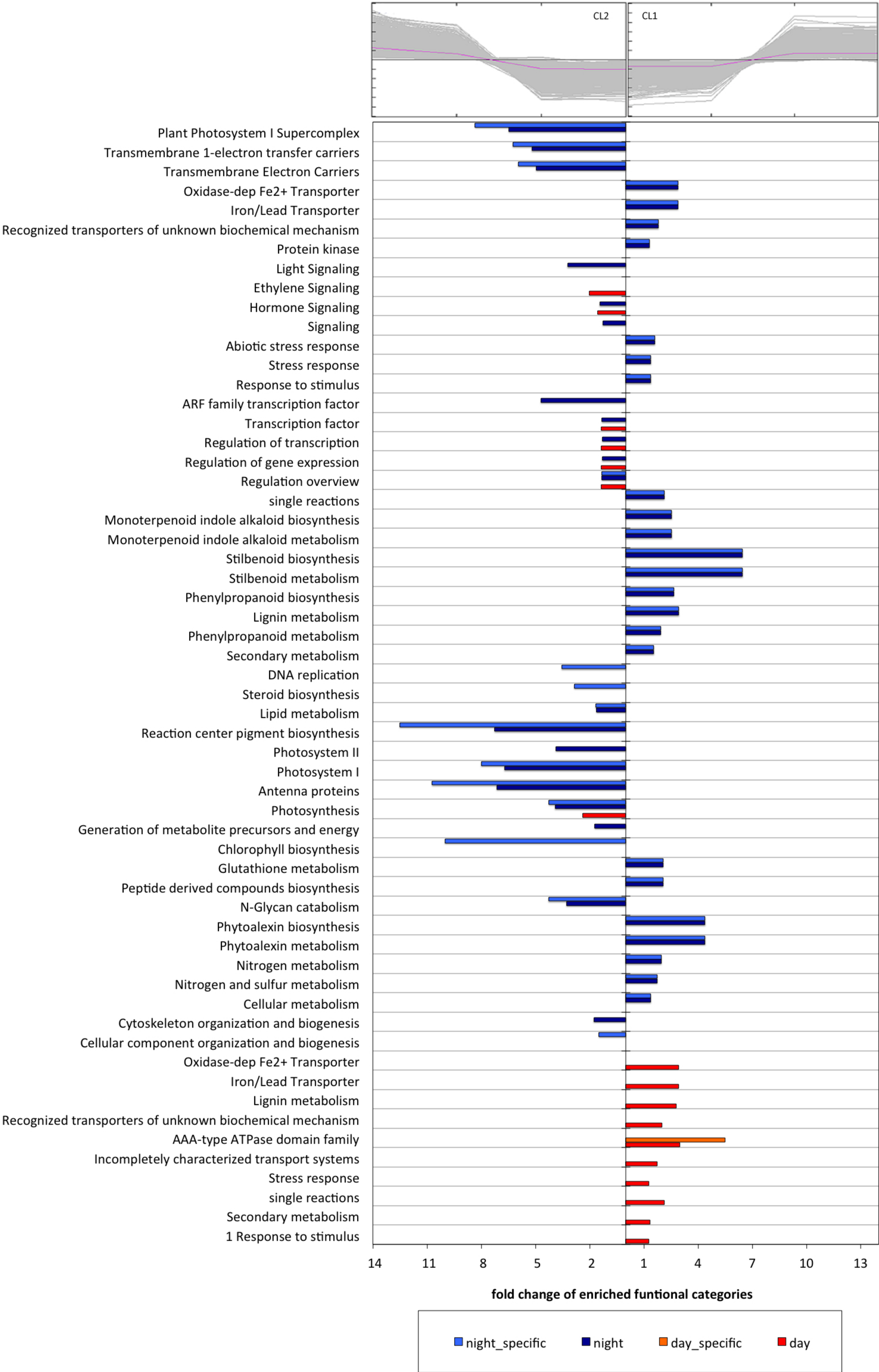

Supplement: Figure S1 — Fold change of enriched functional categories of transcripts allocated to cluster 1 and 2. Categories for all day and night as well as for day and night specific transcript within cluster is illustrated. (PDF) [file pone.0088844.s001.pdf]

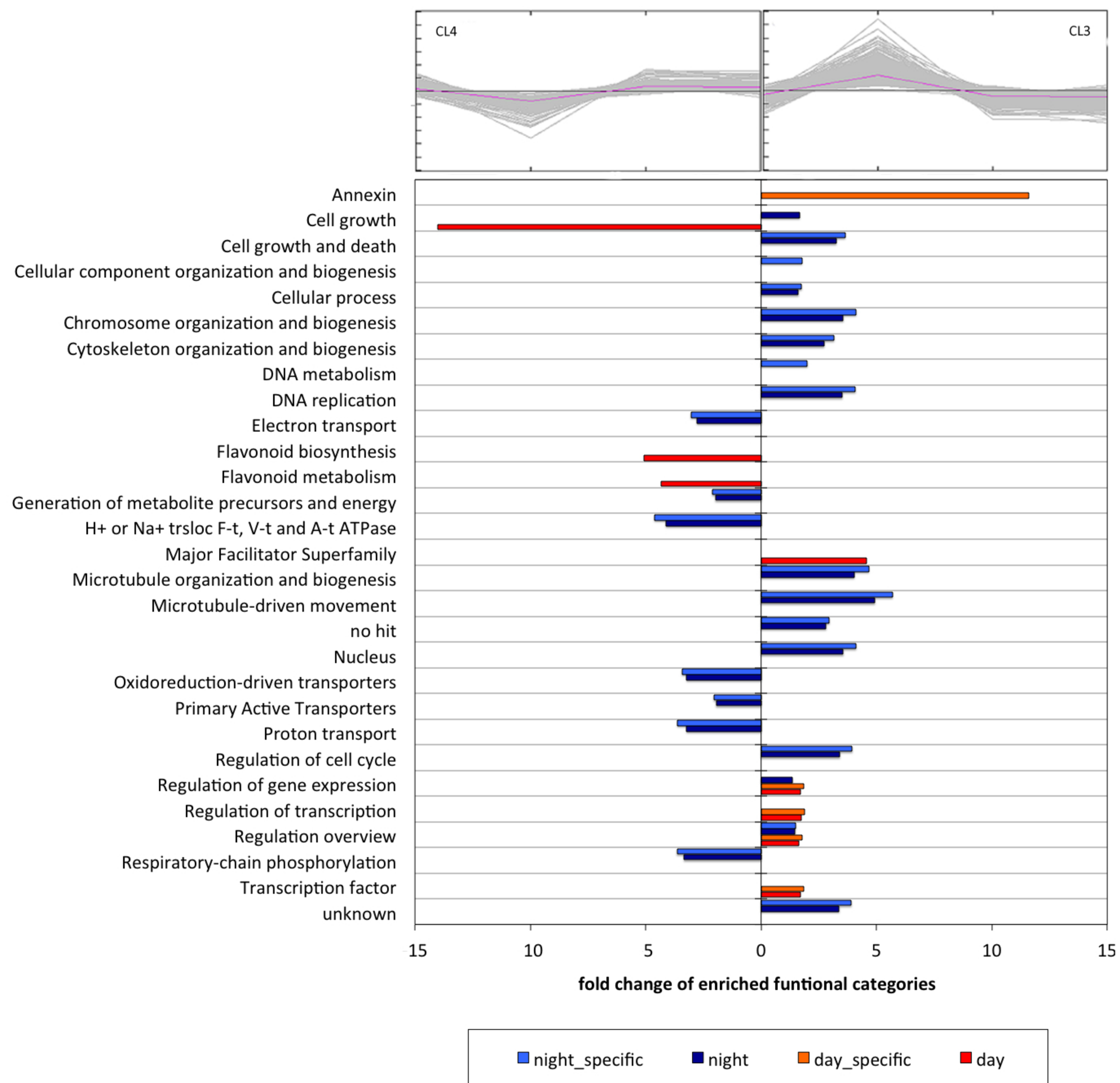

Supplement: Figure S2 — Fold change of enriched functional categories of transcripts allocated to cluster 3 and 4. Categories for all day and night as well as for day and night specific transcript within cluster is illustrated. (PDF) [file pone.0088844.s002.pdf]

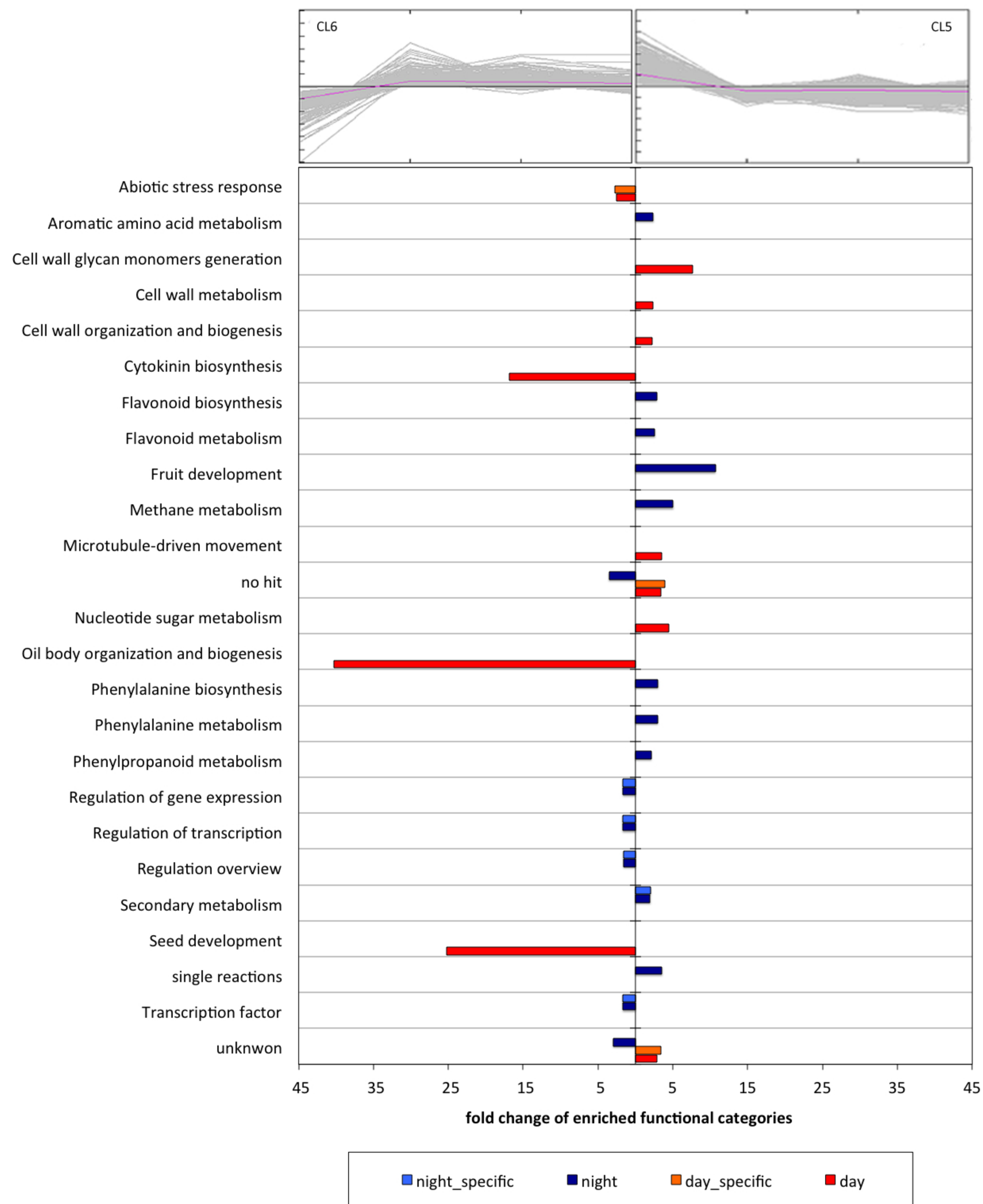

Supplement: Figure S3 — Fold change of enriched functional categories of transcripts allocated to cluster 5 and 6. Categories for all day and night as well as for day and night specific transcript within cluster is illustrated. (PDF) [file pone.0088844.s003.pdf]

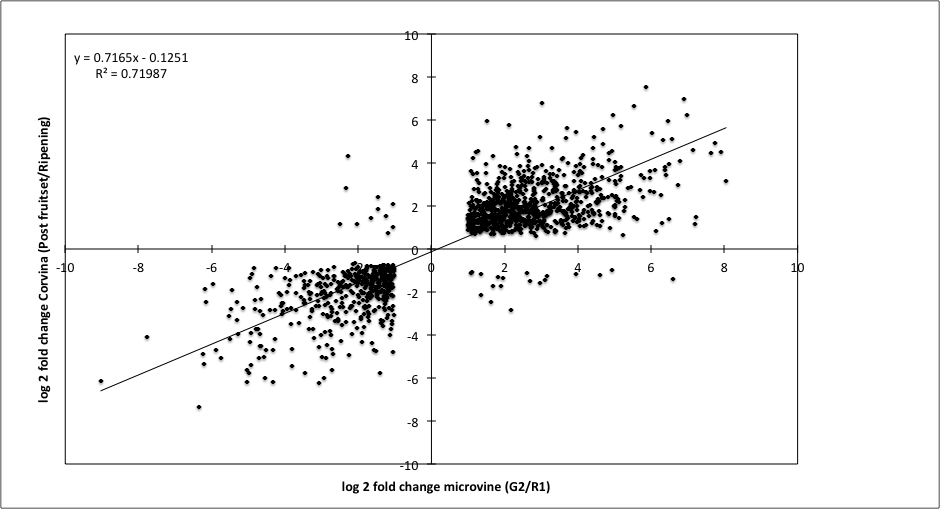

Supplement: Figure S5 — Correlation between genes expression (log2) between green and ripening stages of Corvina L. (Fasoli et al., 2012) and microvine berries. (BMP) [file pone.0088844.s005.bmp]
