# Supplementary material for: Is Transcriptomic Regulation of Berry Development More Important at Night than During the Day?
Source: PLoS One. 2014 Feb 13;9(2):e88844. doi: 10.1371/journal.pone.0088844 (PMC3923830; doi:10.1371/journal.pone.0088844)
Supplement: Figure S4 — Fold change of enriched functional categories of transcripts allocated to cluster 7 and 8. Categories for all day and night as well as for day and night specific transcript within cluster is illustrated. (PDF) [file pone.0088844.s004.pdf]

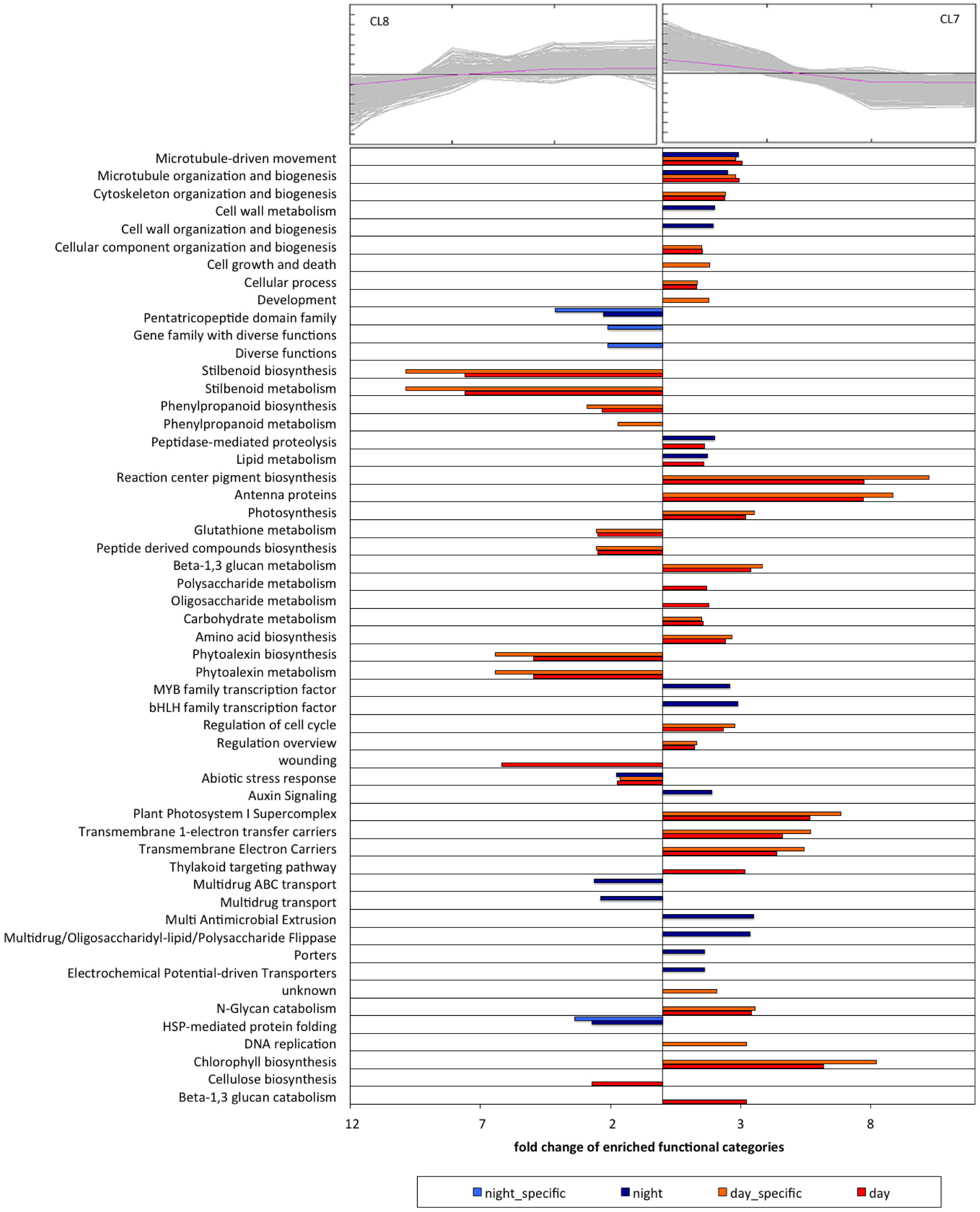
CL8
CL7
Microtubule-driven movement
Microtubule organization and biogenesis
Cytoskeleton organization and biogenesis
Cell wall metabolism
Cell wall organization and biogenesis
Cellular component organization and biogenesis
Cell growth and death
Cellular process
Development
Pentatricopeptide domain family
Gene family with diverse functions
Diverse functions
Stilbenoid biosynthesis
Stilbenoid metabolism
Phenylpropanoid biosynthesis
Phenylpropanoid metabolism
Peptidase-mediated proteolysis
Lipid metabolism
Reaction center pigment biosynthesis
Antenna proteins
Photosynthesis
Glutathione metabolism
Peptide derived compounds biosynthesis
Beta-1,3 glucan metabolism
Polysaccharide metabolism
Oligosaccharide metabolism
Carbohydrate metabolism
Amino acid biosynthesis
Phytoalexin biosynthesis
Phytoalexin metabolism
MYB family transcription factor
bHLH family transcription factor
Regulation of cell cycle
Regulation overview
wounding
Abiotic stress response
Auxin Signaling
Plant Photosystem I Supercomplex
Transmembrane 1-electron transfer carriers
Transmembrane Electron Carriers
Thylakoid targeting pathway
Multidrug ABC transport
Multidrug transport
Multi Antimicrobial Extrusion
Multidrug/Oligosaccharidyl-lipid/Polysaccharide Flippase
Porters
Electrochemical Potential-driven Transporters
unknown
N-Glycan catabolism
HSP-mediated protein folding
DNA replication
Chlorophyll biosynthesis
Cellulose biosynthesis
Beta-1,3 glucan catabolism
12
7
2
3
8
fold change of enriched functional categories
night_specific
night
day_specific
day
